# Gut microbiota modulation via fecal microbiota transplantation mitigates hyperoxaluria and calcium oxalate crystal depositions induced by high oxalate diet

**DOI:** 10.1080/19490976.2025.2457490

**Published:** 2025-01-28

**Authors:** Lingyue An, Shujue Li, Zhenglin Chang, Min Lei, Zhican He, Peng Xu, Shike Zhang, Zheng Jiang, Muhammad Sarfaraz Iqbal, Xinyuan Sun, Hongxing Liu, Xiaolu Duan, Wenqi Wu

**Affiliations:** a Department of Urology, The Second Affiliated Hospital of Guangzhou Medical University, Guangzhou, Guangdong, China; b Guangdong Key Laboratory of Urology, The First Affiliated Hospital of Guangzhou Medical University, Guangzhou, China; c Department of Urology, Minimally Invasive Surgery Center, The First Affiliated Hospital of Guangzhou Medical University, Guangzhou, China; d Department of Urology, Guizhou Provincial People’s Hospital, Guiyang, Guizhou, China

**Keywords:** Hyperoxaluria, high dietary oxalate, gut microbiota, metabolome, fecal microbiota transplantation

## Abstract

Hyperoxaluria, including primary and secondary hyperoxaluria, is a disorder characterized by increased urinary oxalate excretion and could lead to recurrent calcium oxalate kidney stones, nephrocalcinosis and eventually end stage renal disease. For secondary hyperoxaluria, high dietary oxalate (HDOx) or its precursors intake is a key reason. Recently, accumulated studies highlight the important role of gut microbiota in the regulation of oxalate homeostasis. However, the underlying mechanisms involving gut microbiota and metabolite disruptions in secondary hyperoxaluria remain poorly understood. Here, we investigated the therapeutic efficacy of fecal microbiota transplantation (FMT) sourced from healthy rats fed with standard pellet diet against urinary oxalate excretion, renal damage and calcium oxalate (CaOx) crystal depositions via using hyperoxaluria rat models. We observed dose-dependent increases in urinary oxalate excretion and CaOx crystal depositions due to hyperoxaluria, accompanied by significant reductions in gut microbiota diversity characterized by shifts in *Ruminococcaceae_UCG-014* and *Parasutterella* composition. Metabolomic analysis validated these findings, revealing substantial decreases in key metabolites associated with these microbial groups. Transplanting microbes from healthy rats effectively reduced HDOx-induced urinary oxalate excretion and CaOx crystal depositions meanwhile restoring *Ruminococcaceae_UCG-014* and *Parasutterella* populations and their associated metabolites. Furthermore, FMT treatment could significantly decrease the urinary oxalate excretion and CaOx crystal depositions in rat kidneys via, at least in part, upregulating the expressions of intestinal barrier proteins and oxalate transporters in the intestine. In conclusion, our study emphasizes the effectiveness of FMT in countering HDOx-induced hyperoxaluria by restoring gut microbiota and related metabolites. These findings provide insights on the complex connection between secondary hyperoxaluria caused by high dietary oxalate and disruptions in gut microbiota, offering promising avenues for targeted therapeutic strategies.

## Introduction

Hyperoxaluria, including primary and secondary hyperoxaluria, is a disorder characterized by increased urinary oxalate excretion. Primary hyperoxaluria is a rare inherited disorder of oxalate metabolism caused by hepatic enzyme deficiencies, while secondary hyperoxaluria is mainly caused by increased ingestion of oxalate, its precursors or alteration in intestinal microflora. These disorders could lead to recurrent kidney calcium oxalate (CaOx) stones, nephrocalcinosis and eventually end stage renal disease.^1,2^ Although reducing urinary oxalate excretion is pivotal for hyperoxaluria treatment, the drugs and methods currently available for reducing oxalate excretion remain very limited.

Oxalate is mainly excreted through the kidneys. Urinary oxalate is derived from both exogenous sources and endogenous synthesis. Exogenous oxalate originates from the dietary absorption by the intestine. As oxalate is present in many common foods, particularly abundant in spinach, parsley, cocoa, beets and nuts, its intake is unavoidable.^3^ Endogenous oxalate is synthesized in the liver through a pathway which generates glyoxalate as an intermediate molecule, the potential precursors of glyoxalate include hydroxyproline, glycine, glycolate and serine et al .^4^ In addition, as the gut microbiota is capable of degrading oxalate, and oxalate could be absorbed by the intestine, the intestinal tract, together with the liver and kidney, plays a key role in maintaining oxalate homeostasis.

Previous studies have found that high dietary oxalate (HDOx) can disrupt gut microbiota composition and contribute to metabolic disorders.^5–7^ The relation between gut microbiota imbalance caused by long-term HDOx diet and hyperoxaluria remains insufficiently explored. As humans do not produce enzymes to metabolize oxalate, the homeostasis of oxalate in human depend on the balance among its intestinal absorption, excretion and microbial degradation. Oxalate-metabolizing bacterial species in the gut have been speculated to maintain oxalate homeostasis by reducing the amount of dietary oxalate absorption. Oxalate-degrading microorganisms could be classified into two groups: obligate oxalate consumers, such as *Oxalobacter formigenes* (*Oxf*), that require oxalate as a carbon and energy source; and facultative oxalate consumers, including species from the *Lactobacillus, Eggerthella*, and *Streptococcus*, which could degrade oxalate but often exhibit growth inhibition when exposure to oxalate. Notably, transplantation of *Oxf* or other probiotic those with oxalate degrading ability, did not always result in significant reduction in urinary oxalate excretion. Increasing evidences highlight that oxalate degrading capacity of the intestinal microbiota relies on various taxa-composed complex metabolic network, but understanding the network and fully revealing the oxalate-degrading properties of the gut microbiome in hyperoxaluria remain a big challenge to date.^8–10^

Among the potential therapeutic strategies, fecal microbiota transplantation (FMT) has emerged as a promising approach in conditions like *Clostridioides difficile* infection, inflammatory bowel disease, autism, and kidney disease by restoring gut microbiota diversity and functionality.^11–13^ In this study, we investigated the temporal and spatial alterations in gut microbiota, metabolic perturbations, and the therapeutic potential of FMT in mitigating secondary hyperoxaluria, renal injury and CaOx crystal depositions induced by HDOx diet, utilizing a well-established secondary hyperoxaluria rat model. Through an in-depth analysis of the underlying metabolic mechanisms involved in this process, our research seeks to contribute to the development of FMT as a novel and effective preventive and therapeutic strategy for managing hyperoxaluria, as well as renal injury and CaOx crystal depositions induced by high urinary oxalate. The elucidation of these critical aspects holds promising implications for the advancement of precision medicine approaches in the management of oxalate-related disorders and the improvement of clinical outcomes for affected individuals.

## Materials and methods

### Animals

Male Sprague Dawley rats, aged 8 weeks (*n* = 80), were acquired from the Guangdong Medical Laboratory Animal Center and housed in a specific pathogen-free animal facility at the Animal Experimental Center of Guangzhou Medical University Second Affiliated Hospital. The rats were randomly assigned to individual cages with four rats each and were allowed to acclimate for one week with ad libitum access to a standard diet and water. All experimental procedures were performed in compliance with the guidelines of the National Institutes of Health Guide for the Care and Use of Laboratory Animals, and the study was approved by the Second Affiliated Hospital of Guangzhou Medical University Ethics Committee (Approval No. B2022–078).

### Experimental design and sample collection

**Figure 1.**
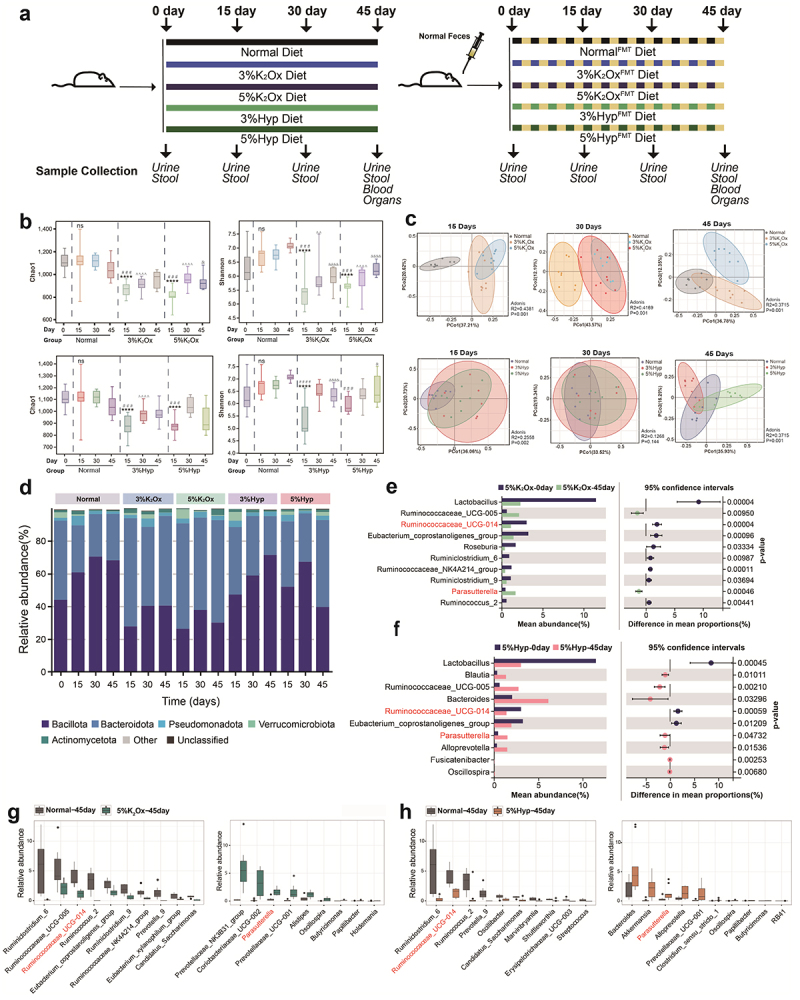
Experimental design and the gut microbiota alteration of rats induced by HDOx.

The experimental design, as depicted in Figure 1a, aimed to induce hyperoxaluria and CaOx crystal depositions in rats through HDOx feeding, consisting of an oxalate diet and an oxalate precursor diet. The oxalate diet included potassium oxalate (K_2_Ox) and hydroxyproline (Hyp) as dietary sources of oxalate and oxalate precursors, respectively.^14,15^ The rats were randomly divided into 10 groups, with each group comprising 8 rats. These groups were designated as follows: standard diet, 3% K_2_Ox, 5% K_2_Ox, 3% Hyp, and 5% Hyp, and the feeding regimen lasted for 45 days. Throughout the experiment, all rats had unrestricted access to sterile water.

(a) Experimental design, the timeline of FMT intervention and sample collection. FMT: Fecal microbiota Transplantation, Hyp: Hydroxyproline, K2Ox: Potassium oxalate. (b) Comparison of alpha diversity between indicated groups, including Chao 1 and Shannon -index. * # ^ &;: *p* < 0.05, * compare to Normal 0 day group, # compare to Normal 15 day group, ^ compare to Normal 30 day group, & compare to Normal 45 day group. (c) The beta diversity of gut microbiota on the timeline showed by PCoA. (d) The relative abundance of HDOx groups on the timeline at the phylum level; (e, f) The differential genera between the Normal group and the HDOx groups at day 45. (g, h) The different genera between the day 0 and day 45 of HDOx groups. ns: no significance. & : *p* < 0.05. ### : *p* < 0.001. **** ,#### ,^^^^ and &&&& : *p* < 0.001.

### Sample collection and fecal microbiota transplantation (FMT)

FMT was performed as previous description.^16,17^ In brief, before HDOx diet exposure, fresh feces from 8-week-old healthy male SD rats that fed with standard pellet diet and tap-water were collected, combined and re-suspended in pre-cooled sterile PBS (200 mg/mL), and centrifuged at 1000 rpm for 10 minutes at 4°C under sterile conditions. The bacterial suspension was mixed with an equal volume of 20% sterile glycerol solution and stored at −80°C until transplantation. The day of feces collection is defined as the Normal 0 day group. Recipient rats were transplanted with 200 μL bacterial suspension by gavage every two days. These groups were respectively labeled as Normal^FMT^, 3% K_2_Ox^FMT^, 5% K_2_Ox^FMT^, 3% Hyp^FMT^, and 5% Hyp^FMT^. During the course of the experiment, fecal and 24-hour urine samples were collected from selected rats at different time points, including at the beginning (day 0) and on days 15, 30, and 45. Fecal samples were rapidly frozen in liquid nitrogen and stored at −80°C for subsequent analysis, while 24-hour urine samples were collected using metabolic cages. After 45 days, all rats were euthanized by intraperitoneal injection of 2% pentobarbital, in accordance with ethical guidelines.

### Renal CaOx crystal depositions and injury evaluation

Kidney tissues were fixed and prepared as 5 μm paraffin-embedded sections. Pizzolato staining was performed to detect CaOx crystal depositions in the kidneys, as previously described. Hematoxylin & Eosin (HE) staining was employed to observe renal injury, and the evaluation of kidney tubular damage was conducted based on lumen dilatation, epithelial necrosis, cast formation, and loss of brush border in HE sections, which were observed using an optical microscope. Two pathologists who blinded to the study design and hypothesis were invited to evaluate the HE slides. Five fields were randomly selected for semi-quantitative scoring, and the percentage of damaged renal tubules was assessed as follows: 0 (no damage), 1 (<25% damage), 2 (25–50% damage), 3 (50–75% damage), and 4 (>75% damage).^18^

### 16S rRNA gene sequencing

Total microbial DNA was extracted from fecal samples using the HiPure Stool DNA Kits (Magen, Guangzhou, China) following the manufacturer’s protocol. The V3-V4 region of the 16S rRNA gene was amplified by PCR, and the cycling conditions were as follows: 95°C for 5 minutes, followed by 30 cycles at 95°C for 1 minutes, 60°C for 1 minutes, 72°C for 1 minutes, and a final extension at 72°C for 7 min, using primers 341F (CCTACGGGNGGCWGCAG) and 806 R (GGACTACHVGGGTATCTAAT). The PCR products were purified, quantified, pooled, and sequenced using an Illumina Novaseq 6000 platform. Microbial burden was quantified with qPCR for the 16S rRNA gene.^19^ Raw reads were processed using FASTP (version 0.18.0) for filtering, merging, and quality control. Noisy sequences of raw tags were filtered under specific filtering conditions, including removing reads containing more than 10% of unknown nucleotides (N), removing reads containing more than 50% of bases with quality (Q-value) < 20, removing adapter contamination) to obtain the high-quality clean tags. Paired reads were overlaped as raw tags using FLASH (version 1.2.11) with a minimum overlap of 10 bp and mismatch error rates of 2%.^20^ Noisy sequences of raw tags were filtered under specific filtering conditions to obtain the high-quality clean tags.^21^ Operational taxonomic units (OTUs) were clustered at ≥ 97% similarity using the UPARSE pipeline (version 9.2.64). All chimeric tags were removed using UCHIME algorithm. The most abundant sequence within each cluster was selected as the representative sequence. The 16S rRNA microbial profiling analysis of the 264 stool samples produced a total number of 21,152,461 reads. The quality and chimaera filtering resulted in 21,134,395 reads, with a 80,054 average per sample. These reads were clustered into 6,261 OTUs. These representative OTU sequences were classified into organisms by a naive Bayesian model using RDP classifier (version 2.2) based on SILVA database (version 138.1), with the confidence threshold value of 0.8. The abundance of each taxonomy was visualized using Krona, and biomarker features in each group were identified using Metastats and LEfSe software.^22,23^

### Microbiota date analysis

The alpha diversity of the gut microbiota was assessed by using the Shannon and Chao1 indices, which were calculated in QIIME (version 1.9.1). Sequence alignment was performed using Muscle (version 3.8.31). The weighted uniform distance matrices were generated to assess beta diversity based on Bray-Curtis distance by using Vegan package (version 2.5.3). The beta diversity of each group, which was assessed by using principal coordinate analysis (PCoA), was generated in the Vegan package (version 2.5.3) and plotted in the ggplot2 package (version 2.2.1). The Adonis test was used to test the microbiome composition between sample. Through the LEfSe software (version 1.0), linear discriminant analysis effect size (LEfSe) was applied to identify the differentially abundant taxa with linear discriminant analysis (LDA) scores exceeding 3.5 within each group. The indicator values for each microbiota within different groups were calculated using the Labdsv package. Subsequently, Indicator Species Analysis was performed to identify differential biomarkers among the groups. Moreover, the MaAslin2 package was applied to identify significant associations between microbiome data and date variable.

PICRUSt analysis was used to identify and quantify the relative abundance of genes associated with oxalate degradation pathways. Specifically, the KEGG pathway analysis of the OTUs was inferred using PICRUSt (version 2.1.4). We specifically searched for genes known to be involved in oxalate degradation pathways, such as CoA:oxalate CoA-transferase, Oxalate decarboxylase, Formate dehydrogenase and Formyl-CoA transferase. Then t-tests were used to compare the relative abundance of these oxalate-degrading genes between different groups.

### Non-targeted metabolome profiling of serum and feces

Non-targeted metabolome profiling was conducted using UHPLC-MS/MS analyses, employing a Vanquish UHPLC system coupled to an Orbitrap Q Exactive^TM^ HF-X mass spectrometer. The UHPLC-MS/MS data underwent processing with Compound Discoverer 3.1, encompassing peak alignment, picking, and quantification of each metabolite. The resulting normalized data were employed for molecular formula prediction and cross-referenced with the mzCloud, mzVault and MassList databases to ascertain qualitative and relative quantitative results.

Principal component analysis (PCA) was applied to describe the data between samples. The Adonis test was used for the statistical analysis of differences between samples. To identify differential metabolites, Orthogonal Projection to Latent Structures-Discriminant Analysis (OPLS-DA) was used to enhance the separation between groups and identify metabolites that contribute significantly to the differences, combined with statistical measures like VIP ≥ 1, P<0.05 to select key differential metabolites. Procrustes analysis was applied to evaluate the agreement between fecal and serum metabolomics profiles, helping to determine how closely these two sets of metabolic data align. Spearman correlation analysis was employed to investigate the relationship between differential bacteria and differential host metabolites in feces or serum.

### Immunohistochemistry (IHC) and immunofluorescence (IF)

IHC and IF procedures were conducted in accordance with established protocols described in previous studies.^18,24^ The following antibodies were employed: anti-ZO1 (1:200 dilution, Abcam), anti-Occludin (1:100 dilution, Cell Signaling Technology), anti-Slc26a6 (1:200 dilution, Abcam), anti-Slc26a3 (1:200 dilution, Abcam), and anti-Slc26a1 (1:200 dilution, Proteintech). IHC sections were visualized using a polarizing microscope (CX31 Olympus, Tokyo, Japan) and a tissue scanner (PathScope 4s, DigiPath, NV, USA). In contrast, IF sections were examined using a fluorescence inverted microscope and an imaging system (Olympus, Japan). The quantification of protein expression was performed using ImageJ software.

### Oxalate measurements of 24-hour urine and fecal stools

To determine the levels of oxalate in 24-hour urine and fecal stools, ion exchange chromatography (Metrohm, Herisau, Switzerland) was employed to measure the 24-hour urine oxalate levels after acidification with 12% HCl.^25^ For fecal oxalate measurement, the methodology from prior studies was adopted.^26,27^ Briefly, stool samples were acidified with 2 M HCl and vortexed for 20 minutes. Supernatant fecal water was collected after centrifuging at 21,000 g at room temperature. The quantification of oxalate in fecal water was carried out using the Oxalate Assay Kit (Abcam, ab196990, Cambridge, UK) following the manufacturer’s instructions.

### Statistical analysis

Statistical analyses were conducted using RStudio (Version 1.4.1106), Graphpad Prism (Version 9.1.1), and SPSS (R23.0.0.0) software. One-way ANOVA was used for the analysis of 24-hour urine oxalate, fecal oxalate, and histological results. Tukey-HSD tests, alpha diversity, and beta diversity analyses were performed, and Welch’s t-tests were used for the relative abundance of differential bacteria. PCA and OPLS-DA were employed for multivariate statistical analysis of the metabolome, and T-test was used to screen differential metabolites. Procrustes analysis and nonparametric Spearman’s rank correlation were utilized to assess the correlation between the microbiota and metabolome data. P-values <0.05 were considered statistically significant.

## Results

### HDOx-induced gut microbiota disturbance in rats

We employed 16S rRNA gene sequencing to comprehensively analyze the gut microbiota and assessed alpha diversity measures, including Chao1 and Shannon index, to evaluate the richness and evenness of microbial communities. The results demonstrated a significant reduction in alpha diversity within the HDOx groups (including K_2_Ox and Hyp) compared to the normal group at each time point (Figure 1b). In addition, significant alterations also were found in the gut microbiota composition in response to the HDOx regimen. Specific bacterial genera exhibited either increased or decreased abundance upon exposure to the HDOx diet. Moreover, our exploration of beta diversity using Bray-Curtis and principal coordinate analysis (PCoA) techniques highlighted conspicuous variations in gut microbiota composition at distinct HDOx diet (Figure 1c, Table S1). Notably, the alpha and beta diversity analysis demonstrated significant dissimilarity between the normal group and the HDOx groups (K_2_Ox and Hyp) at day 15, indicating substantial alterations in the gut microbiota composition at this specific time point.

Interestingly, we observed changes in the abundance of bacterial genera as early as day 15 after initiating the HDOx diet, indicating that the HDOx regimen rapidly influenced the gut microbiota within a short duration. Among the most pronounced changes, the phyla Bacteroidota and Bacillota displayed dynamic alterations on day 15, suggesting an early impact of HDOx on the gut microbiota during the initial phase of the high oxalate diet. At the phylum level, Bacteroidota and Bacillota emerged as the two dominant changing phyla in response to the HDOx regimen, with significant changes mainly observed on day 15 (Figure 1d, Figure S1). Specifically, the relative abundance of Bacteroidota was reduced, while the abundance of Bacillota increased on day 15, potentially influencing the functional capabilities of the gut microbiota in metabolizing and utilizing dietary components, including oxalate. At the genus level, we observed specific bacterial genera that displayed significant down-regulation in the 5% K_2_Ox group on day 45, including *Ruminiclostridium_6, Ruminococcaceae_UCG-005*, *Ruminococcaceae_UCG-014*, *Ruminococcus_2*, *Ruminiclostridium_9*, *Eubacterium_coprostanoligenes_group*, *Ruminococcaceae_NK4A214_group*, *Prevotella_9*, *Eubacterium_xylanophilum_group*, and *Candidatus_Saccharimonas*. In contrast, other genera exhibited noteworthy up-regulation in response to the HDOx regimen, such as *Prevotellaceae_NK3B31_group*, *Coriobacteriaceae_UCG-002*, *Parasutterella*, *Prevotellaceae_UCG-001*, *Alistipes*, *Oscillospira*, *Butyricimonas*, *Papillibacter*, and *Holdemania*. In the 5% Hyp group, the downregulated bacteria were partially the same as in the 5% K_2_Ox group, including *Ruminiclostridium_6*, *Ruminococcaceae_UCG-014*, *Ruminococcus_2*, *Prevotella_9*, and *Candidatus_ Saccharimonas*. In addition, *Oscillibacter*, *Marvinbryantia*, *Shuttleworthia*, *Erysipelotrichaceae_UCG-003*, and *Stapleoccus* were decreased. Similarly, among the upregulated genera, *Parasutterella*, *Prevotellaceae_UCG-001*, *Oscillospira*, *Papillibacter* and *Butyricimonas* were consistent with the 5% K_2_Ox group. The other five increased genera were *Bacteroides*, *Akkermansia*, *Alloprevotella*, *Clostridium_sensu_ stricto_1* and *RB41* (Figure 1e,f). Of note, *Ruminococcaceae_UCG-014* and *Parasutterella* were two main differential genera in the 5% HDOx group on day 45 compared with day 0 (Figure 1g,h). Furthermore, the LefSe, MaAslin and indicator analyses revealed that *Ruminococcaceae_UCG-014* was the key microbiota of the normal group, while the *Parasutterella* was the key microbiota of the HDOx diet groups (Figure S2 a-d).

Notably, significant changes in bacterial abundance were predominantly observed starting from day 15 of the HDOx regimen, indicating a rapid influence on gut microbiota composition within a short timeframe (Figure S2e, f). The altered bacterial genera belonged mostly to the phyla Bacillota and Bacteroidota, with a limited number of genera from other phyla (Table 1). Additional data for the 3% HDOx diet groups were shown in the Figure S3. Compared to the normal group, the relative abundance of *Ruminococcaceae_UCG-014* and *Parasutterella* existed no statistically significant difference in the 3% HDOx diet groups.

**Table 1.** The classification of differential bacteria on day 45.

Phylum	Family	*Genus*
Bacillota	Ruminococcaceae	*Ruminiclostridium_6*^*a*^
Bacillota	Ruminococcaceae	*Ruminococcaceae_UCG-005*
Bacillota	Ruminococcaceae	*Ruminococcaceae_UCG-014*^*a*^
Bacillota	Ruminococcaceae	*Ruminococcus_2*^*a*^
Bacillota	Ruminococcaceae	*Eubacterium_coprostanoligenes_group*
Bacillota	Ruminococcaceae	*Ruminiclostridium_9*
Bacillota	Ruminococcaceae	*Ruminococcaceae_NK4A214_group*
Bacteroidota	Prevotellaceae	*Prevotella_9*^*a*^
Bacillota	Lachnospiraceae	*Eubacterium_xylanophilum_group*
Saccharibacteria	Saccharimonadaceae	*Candidatus_Saccharimonas*^*a*^
Bacillota	Ruminococcaceae	*Oscillibacter*
Bacillota	Lachnospiraceae	*Marvinbryantia*
Bacillota	Lachnospiraceae	*Shuttleworthia*
Bacillota	Erysipelotrichaceae	*Erysipelotrichaceae_UCG-003*
Bacillota	Streptococcaceae	*Streptococcus*
Bacteroidota	Prevotellaceae	*Prevotellaceae_NK3B31_group*
Actinomycetota	Atopobiaceae	*Coriobacteriaceae_UCG-002*
Pseudomonadota	Burkholderiaceae	*Parasutterella*^*b*^
Bacteroidota	Prevotellaceae	*Prevotellaceae_UCG-001*^*b*^
Bacteroidota	Rikenellaceae	*Alistipes*
Bacillota	Ruminococcaceae	*Oscillospira*^*b*^
Bacteroidota	Marinifilaceae	*Butyricimonas*^*b*^
Bacillota	Ruminococcaceae	*Papillibacter*^*b*^
Bacillota	Erysipelotrichaceae	*Holdemania*
Bacteroidota	Bacteroidaceae	*Bacteroides*
Verrucomicrobiota	Akkermansiaceae	*Akkermansia*
Bacteroidota	Prevotellaceae	*Alloprevotella*
Bacillota	Clostridiaceae_1	*Clostridium_sensu_stricto_1*
Actinomycetota	Pyrinomonadaceae	*RB41*

^a^indicates that the genus was downregulated simultaneously in the 5% K_2_Ox and 5% Hyp group, ^b^ indicates that the genus was upregulated simultaneously in the 5% K_2_Ox and 5% Hyp group.

Moreover, the alpha diversity of the gut microbiota in the HDO_X_ diet groups was observed to be lower on day 45 compared to the normal group (Figure S3c). Additionally, the beta diversity analysis revealed significant differences in gut microbiota composition among the normal, 3% HDOx, and 5% HDOx groups (Figure 1c). Notably, as the concentration of K_2_Ox and Hyp increased, there was a decrease in the relative abundance of Bacillota and an increase in Bacteroidota (Figure S3d). These changes were more pronounced in the 5% HDOx diet groups, prompting us to focus on this dosage for subsequent analyses.

### HDOx-induced metabolites disturbance in feces and serum

**Figure 2.**
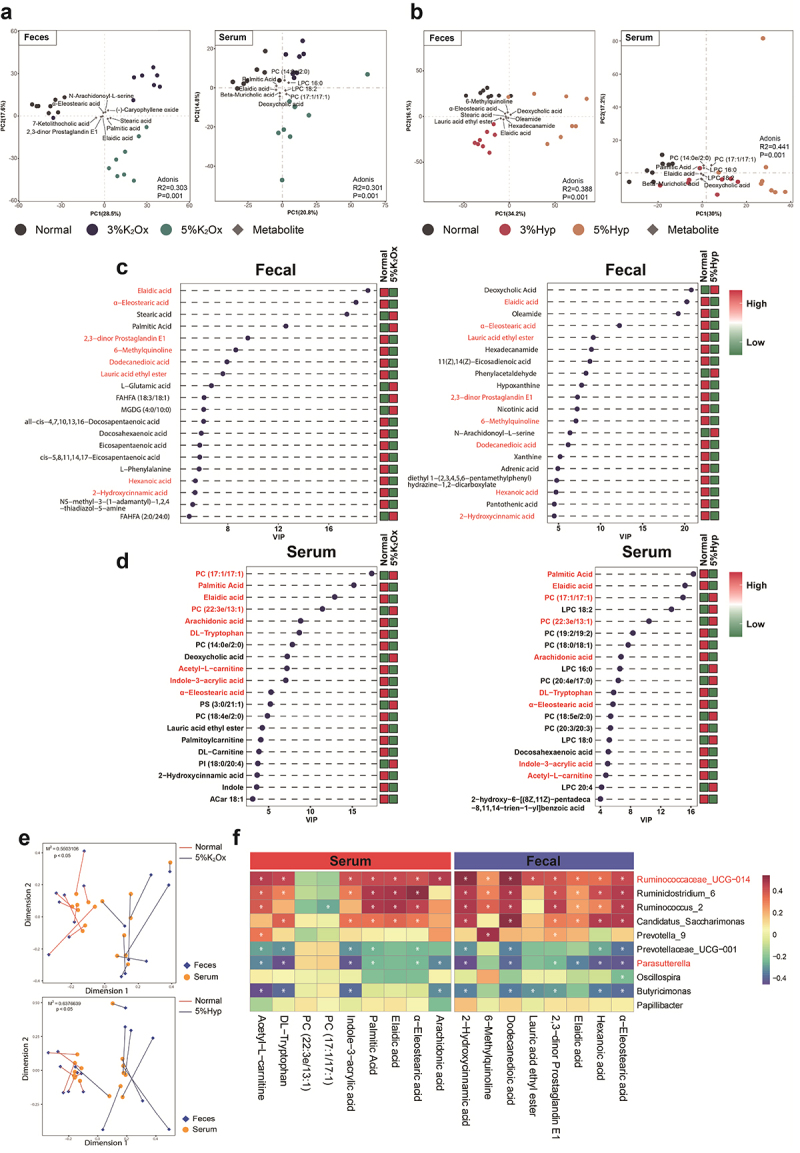
The metabolic changes induced by HDOx.

In the context of HDOx-induced metabolite disturbances, we conducted an examination of the metabolic changes at day 45. The results of the PCA analysis revealed that fecal metabolites exhibited higher heterogeneity compared to serum metabolites. Additionally, the differences in fecal metabolites appeared to be dose-dependent in the K_2_Ox and Hyp-treated groups, with particularly notable variations between the 5% concentration group and the normal group. Notably, several key metabolites, including α-Eleostearic acid, Stearic acid, and Elaidic acid in fecal samples, as well as Deoxycholic acid, Palmitic acid, and Elaidic acid in serum samples, were consistently found in all HDOx-treated groups. These metabolites are likely to play crucial roles in the oxalate metabolic disorders induced by the HDOx regimen (Figure 2a,b, Table S2).

(a, b) Separation of fecal and serum metabolome between the Normal group and K_2_Ox groups (a), Hyp groups (b) at day 45 revealed by PCA. The main metabolites that contribute to the separation are indicated by diamonds. (c, d) The top 20 differential metabolites in feces and serum. Differential metabolites were filtered for VIP ≥ 1 based on the OPLS-DA and T-test *p* < 0.05, the red represents common metabolites. (e) Procrustes analysis of fecal and serum metabolome. Serum and fecal samples are shown as orange circles and blue squares, respectively, serum and stool samples from the same rat are connected by red (Normal) and dark blue (5% K_2_Ox or 5% Hyp) lines. (f) The correlation between common differential bacteria (genus level) and common differential metabolites in feces and serum. * indicates a significant correlation.

Furthermore, we compared the metabolic alterations in feces and serum between the normal and 5% HDOx diet groups. In total, 164 differentially changed metabolites (DCMs) in feces were identified in the 5% Hyp group compared to the normal group, while 240 DCMs were identified in the 5% K_2_Ox group. Interestingly, 99 metabolites were shared between the 5% Hyp and 5% K_2_Ox groups, indicating common metabolic disturbances induced by these two high oxalate concentrations (Figure S4a). Similarly, in serum samples, 103 DCMs were observed in the 5% Hyp group compared to the normal group, while 71 DCMs were identified in the 5% K_2_Ox group. Among these, 42 metabolites were shared between the 5% Hyp and 5% K_2_Ox groups (Figure S4b).

Further analysis of the top 20 DCMs in feces and serum showed that 8 DCMs were shared in both the 5% K_2_Ox and 5% Hyp groups, and 9 DCMs were common to both feces and serum. Notably, Elaidic acid and α-Eleostearic acid were the only two metabolites that significantly decreased in both the 5% K_2_Ox and 5% Hyp groups compared to the normal group, indicating their potential role as key biomarkers of HDOx-induced metabolic changes. Moreover, 2-Hydroxycinnamic acid and Lauric acid ethyl ester were significantly down-regulated in both feces and serum, except for the serum of the 5% Hyp group. Additionally, Palmitic Acid significantly increased in the feces of the 5% K_2_Ox group, whereas it showed a notable decrease in the serum of both the 5% K_2_Ox and 5% Hyp groups (Figure 2c,d). These findings provide important insights into the specific metabolites that are affected by the HDOx regimen and their potential contributions to oxalate-relatedmetabolic disturbances.

### The crosstalk between HDOx-induced gut microbiota disorder and metabolites disturbance

In light of the close association between metabolite disturbances and gut microbiota disorders, we proceeded to investigate the interplay between HDOx-induced metabolite disturbances and alterations in the gut microbiota. Employing Procrustes analysis, we observed significant associations between the fecal and serum metabolomes in the normal group compared to the 5% K_2_Ox (M2 = 0.55, *p* < 0.05) or 5% Hyp (M2 = 0.64, *p* < 0.05) treated groups (Figure 2e). Subsequent correlation analysis between metabolites and gut bacteria revealed substantial correlations between differential bacterial genera and metabolites in both feces and serum, with notable emphasis on the relationship between *Ruminococcaceae_UCG-014* and *Parasutterella* (Figure 2f).

A significant positive correlation was observed between *Ruminococcaceae_UCG-014* and α-Eleostearic acid, as well as Elaidic acid, in both feces and serum. These two key metabolites exhibited decreases in abundance in both the 5% K_2_Ox and 5% Hyp groups. Additionally, *Ruminococcaceae_UCG-014* showed a positive correlation with 2-Hydroxycinnamic acid, which was another important decreased metabolite, except in the serum of the 5% Hyp group. Conversely, *Parasutterella* displayed a negative association with α-Eleostearic acid in both feces and serum, as well as Elaidic acid and 2-Hydroxycinnamic acid in feces. Moreover, *Ruminococcaceae_UCG-014* exhibited a positive correlation with Palmitic acid in serum, while *Parasutterella* showed a negative correlation with it. Notably, Palmitic acid was significantly up-regulated in the feces of the 5% K_2_Ox group but decreased in the serum of both the 5% K_2_Ox and 5% Hyp groups. These findings suggest that *Ruminococcaceae_UCG-014* and *Parasutterella* may be the two main bacterial genera significantly correlated with differential metabolites in both feces and serum.

### FMT attenuated HDOx-induced urinary oxalate excretion and renal CaOx crystal depositions

**Figure 3.**
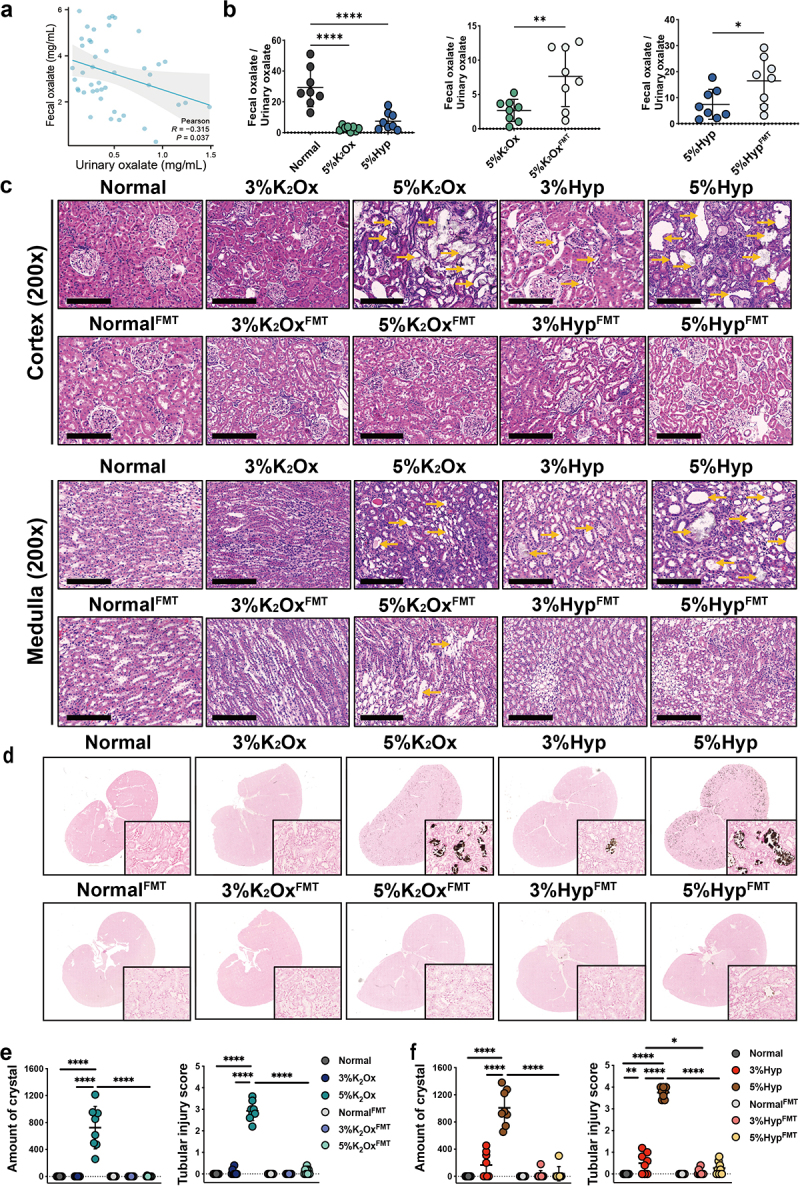
The effects of FMT on HDOx-induced renal injury and CaOx crystal depositions.

We further explored the effects of FMT on HDOx-induced fecal and urinary oxalate excretion, as well as renal injury and CaOx crystal depositions. As shown in Figure 3a, there was a negative correlation between urinary oxalate concentration and fecal oxalate concentration. Compared to the normal group, the ratio of Fecal oxalate/Urinary oxalate was markedly decreased in the groups fed with HDOx, whereas FMT treatment could significantly reverse this decrease (Figure 3b). As FMT treatment increased the fecal oxalate but markedly decreased the urinary oxalate, this opposite alteration after FMT suggested that FMT may relieve urinary oxalate excretion by increasing intestinal oxalate secretion (Figure S5a). In addition, as observed earlier, rats exposed to 5% K_2_Ox and 5% Hyp exhibited severe renal tubular lumen dilatation, epithelial necrosis, cast formation, and brush border loss, indicative of significant renal injury. Conversely, 3% Hyp resulted in only slight dilation of tubular lumens, while 3% K_2_Ox showed minimal effects on renal injury, demonstrating the influence of HDOx on renal health is dose-dependent (Figure 3c).

(a) The correlation analysis between the Fecal oxalate and Urinary oxalate of all the samples at day 45. (b) The comparison of Fecal oxalate / Urinary oxalate ratio between indicated groups. (c) Renal cortical and medulla damage caused by HDOx and the ameliorating effect of FMT. (d) The effects of FMT on HDOx-induced CaOx crystal depositions in rat kidneys. (e, f) The CaOx crystal number and tubular injury score in different groups. Bar = 100 μm, yellow arrows identify areas of significant tubular injury. * P<0.05, ** P<0.01, **** P<0.001.

HDOx, particularly in the 5% K_2_Ox and 5% Hyp groups, significantly increased urinary oxalate excretion and kidney CaOx crystal depositions in rats. However, the administration of FMT had a remarkable mitigating effect on these alterations induced by HDOx (Figure S5b-e, Figure 3b,d and Table 2). Moreover, FMT demonstrated a substantial protective effect against HDOx-induced renal injury (Figure 3e,f). These findings highlight the potential of FMT as a therapeutic intervention to counter the adverse effects of HDOx on the kidneys and urinary system.

**Table 2.** The 24-hr urinary oxalate excretion on the timeline in different groups.

Groups	Mean ± Standard deviation (mg/24 h)			
	0 day	15th day	30th day	45th day
Normal	2.78 ± 1.40	3.24 ± 1.91	3.89 ± 1.44	4.02 ± 0.85
3%K_2_Ox	2.90 ± 1.50	4.07 ± 1.21	5.24 ± 1.13	5.67 ± 1.12
5%K_2_Ox	3.12 ± 1.48	8.95 ± 2.88^* a c^	10.50 ± 2.67^* a c^	10.95 ± 3.39^* a c^
3%Hyp	2.64 ± 1.83	5.94 ± 1.73*	6.47 ± 1.18*	6.39 ± 1.29*
5% Hyp	3.44 ± 2.15	10.18 ± 3.09^* b c^	12.69 ± 2.67^* b c^	13.79 ± 3.37^* b c^
Normal^FMT^	3.55 ± 1.30	4.16 ± 1.20	4.25 ± 1.15^b^	4.48 ± 1.43
3%K_2_Ox^FMT^	3.43 ± 0.92	4.28 ± 1.56	4.82 ± 1.38	4.75 ± 1.18
5%K_2_Ox^FMT^	3.20 ± 1.67	4.77 ± 1.54	5.84 ± 1.54*	7.04 ± 2.17*
3%Hyp^FMT^	3.43 ± 0.92	4.28 ± 1.56	4.82 ± 1.38	4.75 ± 1.18
5% Hyp^FMT^	3.65 ± 1.88	5.18 ± 2.11	5.27 ± 1.38	7.33 ± 2.56*

Compared to the same days: * *p* < 0.05 compared to the Normal group, ^a^ *p* < 0.05 compared to the 3% K_2_Ox group, ^b^ *p* < 0.05 compared to the 3% Hyp group, ^c^ *p* < 0.05 compared to the corresponding FMT group.

### FMT partially corrected HDOx-induced gut microbiota disorder and metabolic disturbance

**Figure 4.**
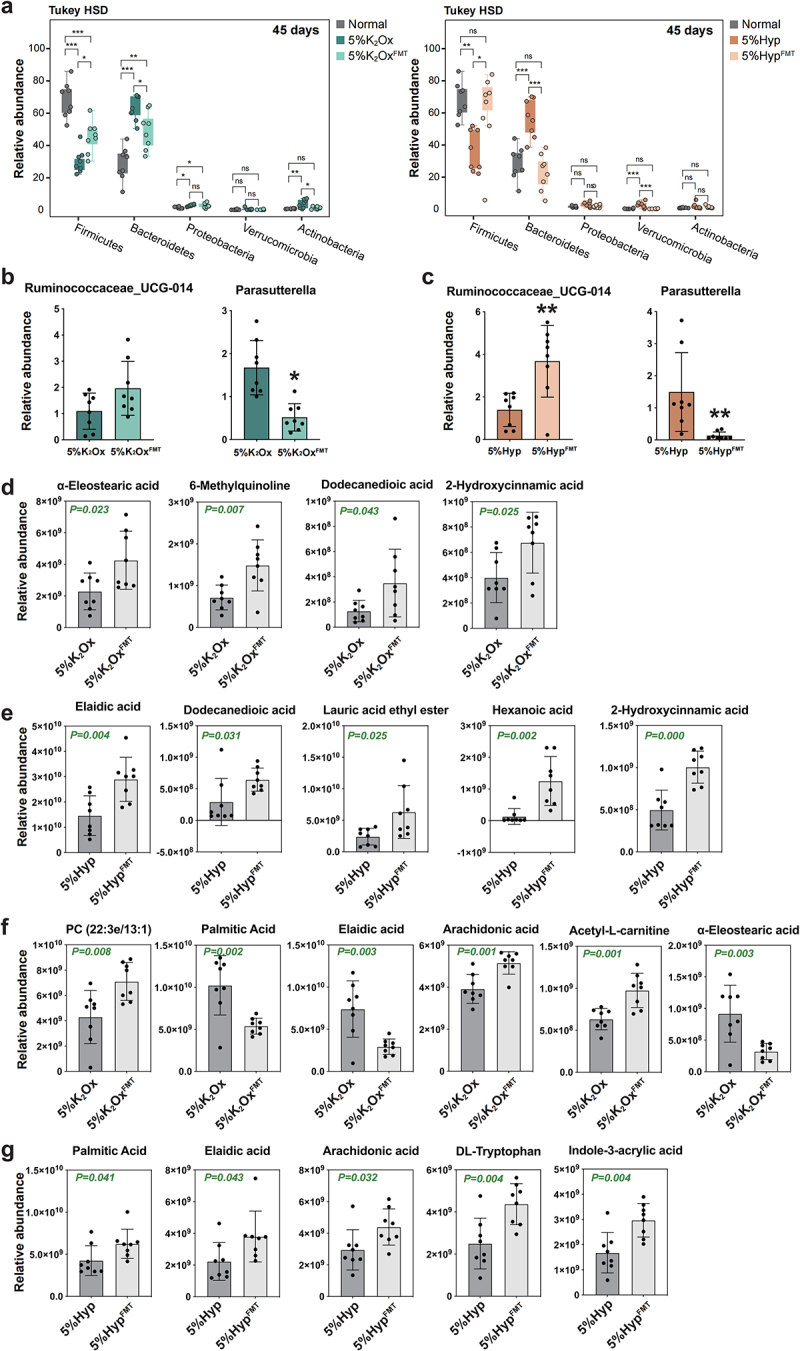
The effects of FMT on gut microbiota and metabolism.

To elucidate the underlying mechanism of FMT in ameliorating HDOx-induced urinary oxalate excretion and renal CaOx crystal depositions, we investigated the effects of FMT on HDOx-induced gut microbiota and metabolic disorders. The results revealed a significant restoration in both the alpha and beta diversity of gut microbiota in the HDOx-treated groups following FMT (Figure S6). Moreover, the relative abundance of Bacillota and Bacteroidota, which was perturbed by HDOx, showed notable recovery after FMT treatment (Figure 4a). Furthermore, the differential genera that were induced by 5% K_2_Ox or 5% Hyp, including *Ruminococcaceae_UCG-014* and *Parasutterella*, exhibited substantial recovery after FMT treatment (Figure 4b,c, and Figure S7). Similarly, most of the HDOx-induced DCMs in both feces and serum were significantly restored by FMT treatment. Notably, among the 8 common fecal DCMs mentioned earlier, 4 DCMs, including α-Eleostearic acid, 2-Hydroxycinnamic acid, 6-Methylquinoline, and Dodecanedioic acid, showed significant elevation in the 5% K_2_Ox^FMT^ group compared to the 5% K_2_Ox group (Figure 4d). Additionally, 5 DCMs, namely Elaidic acid, Dodecanedioic acid, Lauric acid ethyl ester, Hexanoic acid, and 2-Hydroxycinnamic acid, were significantly increased in the 5% Hyp^FMT^ group compared to the 5% Hyp group (Figure 4e). Similarly, in the 9 common serum DCMs mentioned above, 6 DCMs in the 5% K_2_Ox^FMT^ group and 5 DCMs in the 5% Hyp^FMT^ group were significantly restored by FMT (Figure 4f,g). These findings underscore the potential of FMT to partially correct the gut microbiota disorder and metabolic disturbances induced by HDOx, thereby mitigating urinary oxalate excretion and renal CaOx crystal depositions.

(a) The relative abundance of differential bacteria at the phylum level was repaired by FMT at day 45. (b, c) The relative abundance of two key differential bacteria after FMT at day 45. (d, e) The fecal differential metabolites alteration between the HDOx groups and FMT-treated groups. (f, g). The serum differential metabolites alteration between the HDOx groups and FMT-treated groups. ns indicates no statistical difference, * P < 0.05, ** P < 0.01, *** P < 0.005.

### FMT regulated oxalate metabolism via repairing the intestinal barrier and oxalate transporters

In our investigation of FMT and its impact on oxalate metabolism, we initially analyzed the expressions of oxalate degradation enzymes in the gut microbiota, specifically CoA:oxalate CoA-transferase, Oxalate decarboxylase, Formate dehydrogenase, and Formyl-CoA transferase. Surprisingly, no significant differences were found in the expression levels of these enzymes among the different experimental groups (Figure S8). This suggested that FMT’s restoration of oxalate metabolism was not attributed to changes in the oxalate degradation capacity of the gut microbiota.

**Figure 5.**
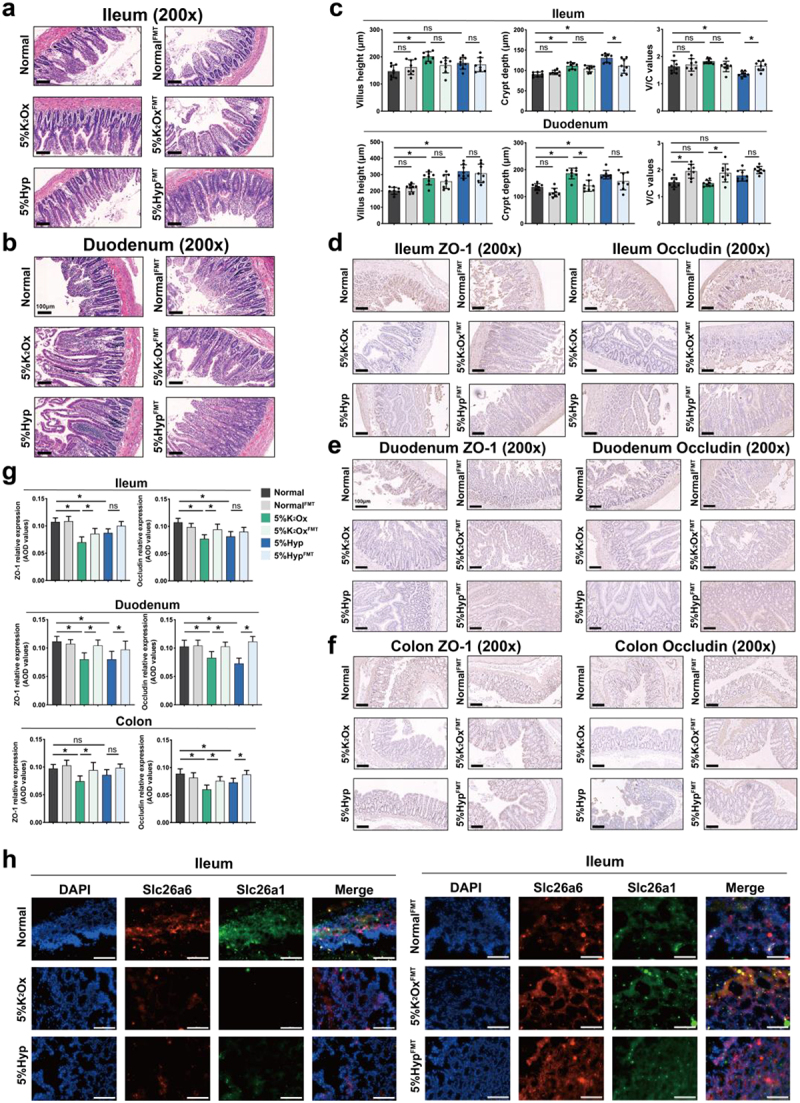
The effects of FMT on HDOx-induced intestinal barrier injury and oxalate excretion.

We then focused on the integrity of the intestinal barrier, as it plays a crucial role in oxalate absorption. The results revealed that rats exposed to 5% K_2_Ox and 5% Hyp exhibited notable increases in the villus height and crypt depth of the ileum, indicative of intestinal injury. Though the V/C value represented no significant difference between the normal and 5% K_2_Ox group, it significantly decreased in the 5% Hyp group compared to the normal group. These alterations induced by K_2_Ox or Hyp were effectively reversed by FMT treatment (Figure 5a–c). Besides, FMT treatment could obviously attenuated HDOx-induced decreasing of ZO-1 and Occludin expressions in ileum and duodenum (Figure 5d,e). In colon, though 5% Hyp treatment showed no obvious effect on ZO-1 expression, FMT treatment significantly reversed HDOx-induced decrease of Occludin expression, as well as decreased ZO-1 expression in 5% K_2_Ox group (Figure 5f,g). These findings suggest that FMT treatment can successfully restore the intestinal barrier injury caused by HDOx. Furthermore, FMT treatment also could significantly restore the decrease of oxalate transporters’ expressions, including Slc26a6 and Slc26a1, induced by HDOx (Figure 5h and Figure S9).

(a – c) The villus height, crypt depth, and V/C value alteration of rat intestine among different groups are observed by HE staining. (d – g) The relative expression of barrier function (ZO-1 and Occludin) in the different intestines is measured by IHC staining. (h) The IF staining of ileum oxalate transporters (Slc26a6 and Slc26a1) among different groups. ns: no significance. *p < 0.05. Bar = 100 μm.

## Discussion

In this study, we investigated the effects of a high dietary oxalate (HDOx) intake and fecal microbiota transplantation (FMT) on gut microbiota, metabolic balance, oxalate metabolism, and CaOx crystal depositions in rat kidneys. Our results demonstrate that FMT treatment effectively attenuated HDOx-induced urinary oxalate excretion, renal injury and CaOx crystal depositions by restoring oxalate metabolism through the repair of the intestinal barrier and regulation of oxalate transporters. These beneficial effects were achieved by addressing the HDOx-induced gut microbiota disorder and metabolic disturbance.

The role of dietary intake of oxalate and its precursors in determining urinary oxalate excretion and hyperoxaluria, as well as kidney stone risk, is well established. Previous studies have shown that increased oxalate intake leads to a corresponding increase in urinary oxalate excretion, with percentages ranging from 25% to 53% in response to higher oxalate intake.^28^ While it is known that diet plays a crucial role in shaping the composition of the gut microbiome, the specific impact of gut microbiota profiles induced by dietary patterns, particularly high oxalate diets, on secondary hyperoxaluria has remained poorly understood.

Though secondary hyperoxaluria is a key reason for recurrent kidney CaOx stone and mainly affected by HDOx diet intake or intestinal microflora alteration, previous researches have provided conflicting findings regarding the impact of diet on the gut microbiome composition in individuals with kidney stones. Kim et al. reported that the gut microbiome profile in kidney stone formers is not affected by diet.^29^ Ticinesi et al. also demonstrated dietary habit, except calcium intake, is apparently not involved in the kidney stone-associated abnormalities of the gut microbiota composition.^30^ In contrast, significant differences were found in the dietary patterns of individuals with kidney stone and non-kidney stone, and dietary pattern could affect the homeostasis of gut microbiota.^31^ Here, our results revealed that HDOx diet not only reduces the gut microbiome alpha-diversity, but also induced significant alteration of the beta diversity, suggesting that HDOx diet could efficiently modulate the abundance and composition of gut microbiota. In detail, Bacteroidota and Bacillota, which are the two main phyla that significantly different between the healthy controls and kidney stone patients,^8,31^ were the two dominant changing phyla induced by HDOx diet. On the genus level, most of the differential genera also belonged to Bacteroidota and Bacillota. Remarkably, FMT treatment effectively corrected the abnormal abundance of Bacillota and Bacteroidota, as well as the increased urinary oxalate excretion and renal CaOx crystal depositions induced by the HDOx diet, highlighting the critical roles of Bacillota and Bacteroidota in regulating secondary hyperoxaluria and CaOx crystal depositions. More importantly, our findings provide valuable insights into the potential impact of dietary patterns, particularly the HDOx diet, on the gut microbiome profile and its association with secondary hyperoxaluria. These findings contribute to a better understanding of the complex interplay between diet, gut microbiota, and secondary hyperoxaluria, laying the groundwork for further research in this area.

Previous researches have focused on the gut microbiota with oxalate-degrading activity, especially on *Oxalobacter formigenes* (*Oxf*), which remains one of the most efficient oxalate-degrading bacterium known to date. However, the role of *Oxf* in kidney stone formation remains controversial, which may be due to various reasons, such as the study population, lifestyle, dietary habits, and kidney stone disease status, all of which may affect the gut microbiota.^8,9,32^ In addition, the oxalate degradation enzymes, including CoA:oxalate CoA-transferase and Formyl-CoA transferase, also are crucial for the oxalate-degrading activity of *Oxf*. Interestingly, using PICRUSt analysis, we predicted that HDOx treatment did not significantly affect the abundance of these key enzymes, suggesting that the oxalate-degrading activity might not be directly influenced by the high oxalate dietary pattern employed in our study. Apart from *Oxf*, other microbiota that play active roles in oxalate metabolism, including *Lactobacillus, Bifidobacterium, Muribaculace*ae, *Ruminococcus*, *Oscillospira, Enterococcus, Clostridium, Eggerthella, Providencia, Streptococcus* and *Leuconostoc genera*, were also found to be associated with oxalate excretion and kidney stone disease. Consistent with prior reports, we observed significant changes in the abundance of *Prevotellaceae, Prevotella_9, Ruminococcus, Oscillospira* and *Parasutterella* after HDOx treatment. Notably, *Prevotellaceae_UCG-001*, a bacterial genus widely considered to be probiotic, showed a significant increase in abundance after HDOx treatment, consistent with the findings reported by Zhou et al .^31^ Our results suggest that the capacity for oxalate degradation is shared by various taxa within a complex metabolic network, rather than being dependent on a single specific player or a limited number of species. Notably, to explore the reason for 3% groups showed much less urinary oxalate excretion and kidney damage than 5% groups, we found that compared to the normal group, the abundance of *Ruminococcaceae_UCG-014* was significantly decreased and *Parasutterella* abundance obviously increased in the 5% HDOx diet groups, whereas no significant differences were found between the normal group and 3% HDOx diet groups. These differences may result in distinct effects on the urinary oxalate excretion, renal injury and CaOx crystal depositions, indicating the key roles of *Ruminococcaceae_UCG-014* and *Parasutterella* in the regulation of hyperoxaluria. Moreover, the HDOx-induced alterations in the gut microbiota mentioned above, along with their associated differential metabolites, were effectively restored by FMT treatment, suggesting that these specific gut microbiotas may play key roles in the regulation of secondary hyperoxaluria induced by a high oxalate dietary pattern.

As the gut microbiome’s primary communicators, metabolites play crucial roles in regulating disease processes by interacting with various host factors or activating related signaling pathways. In our present study, HDOx treatment significantly induced disturbances in fecal and serum metabolites, and a close correlation between differential metabolites and differential genera, specifically the *Ruminococcaceae_UCG-014* and *Parasutterella*, was found. Specifically, α-Eleostearic acid and Elaidic acid, which were correlated with *Ruminococcaceae_UCG-014*, were markedly decreased in feces and serum after HDOx treatment. Additionally, other key HDOx-induced differential metabolites associated with *Ruminococcaceae_UCG-014* and *Parasutterella*, including Elaidic acid, 2-Hydroxycinnamic acid, Arachidonic acid, Acetyl-L-carnitine, and Palmitic acid possessed antioxidant and/or anti-inflammatory activities. These findings suggest that the gut microbiome and its metabolites might play pivotal roles in modulating the balance of oxidative stress and immune responses during hyperoxaluria-induced renal damage and CaOx stone formation. For instance, α-Eleostearic acid, a member of the conjugated linolenic acid family, has been shown to inhibit inflammation and regulate anti-oxidant effect in inflammatory bowel disease.^33^ Similarly, 2-Hydroxycinnamic acid is a cinnamic acid derivative with antioxidant activity, while Arachidonic acid is known for its anti-inflammatory properties. Furthermore, Acetyl-L-carnitine possesses strong antioxidant activity.^34–36^ Of note, these differential metabolites mentioned above, along with *Ruminococcaceae_UCG-014* and *Parasutterella*, were significantly restored by FMT. Considering the critical role of oxidative stress in the regulation of CaOx stone formation, the antioxidant activities of *Ruminococcaceae_UCG-014* and *Parasutterella*-produced metabolites may represent key potential mechanisms by which the gut microbiota modulates HDOx-induced hyperoxaluria and CaOx crystal depositions, though their exact roles require further investigation. While certain metabolites identified in our study lack well-established biological activities, such as Lauric acid ethyl ester, its precursor, lauric acid, has been reported to inhibit bacterial biofilm formation when combined with arginine.^37^ Bacterial biofilms have been implicated in promoting crystal aggregation in urine and are considered influential in stone formation.^38^ Thus, it is plausible that Ethyl laurate or its associated metabolites may influence CaOx stone formation by influencing bacterial biofilms synthesis. Considering that oxidative damage is recognized as one of the key mechanisms contributing to hyperoxaluria – induced renal injury and CaOx stone formation, the antioxidant activity displayed by many of the identified metabolites in our study suggests their potential involvement in the regulation of this process. Our finding provides valuable insights into the impact of a high oxalate dietary pattern on gut microbiota and its consequential metabolic changes, which, in turn, influence hyperoxaluria and CaOx crystal depositions. Notably, the identified metabolites, particularly those exhibiting antioxidant properties, may play a substantial role in this context. However, further investigations are warranted to comprehensively understand the specific mechanisms underlying the interactions between these metabolites and gut microbiota, thereby influencing hyperoxaluria.

FMT has emerged as a promising therapeutic approach for various diseases, including *Clostridioides difficile* infection, intestinal inflammation, colitis, obesity, and stroke.^39–43^ More recently, several studies have demonstrated its potential in modifying urine chemistry risk factors for kidney stone disease and inhibiting CaOx crystal depositions in the kidneys through the restoration of gut microbial balance.^44,45^ In line with these findings, our results revealed that FMT not only could partially restore HDOx-induced gut microbiota disorder and metabolites disturbance, but also obviously attenuate HDOx-induced urinary oxalate excretion, renal injury and CaOx crystal depositions.

Oxalate-metabolizing bacterial species in the gut could sustain oxalate homeostasis via reducing the amount of dietary oxalate absorbed and sequestering circulating oxalate into the digestive tract, thus decreasing urinary oxalate excretion and attenuating renal injury, as well as CaOx crystal depositions. In an effort to elucidate the mechanisms underlying FMT’s effects on oxalate metabolism, we investigated the expressions of oxalate degradation enzymes in the gut microbiota and found that FMT had no significant impact on these enzymes’ expressions. Moreover, FMT did restore certain oxalate-metabolizing bacterial species, such as *Ruminococcus* and *Oscillospira*, suggesting their effects might be mediated by other mechanisms. This finding prompted us to explore the effects of FMT on the integrity of the intestinal barrier and the expressions of oxalate transporters, which play pivotal roles in regulating oxalate absorption and excretion.

The excretion of urinary oxalate is influenced by the absorption of dietary oxalate, together with its secretion into the intestine, and degradation by the gut microbiota. Dietary oxalate absorption primarily occurs through two pathways: the paracellular pathway mediated by tight junctions and the transcellular pathway mediated by oxalate transporters, specifically the solute-linked carrier (Slc)-26 family.^46,47^ In the present study, although there were slight differences in the effects of 5% K_2_Ox and 5% Hyp on intestinal barrier integrity and tight junction protein expressions; however, FMT treatment notably attenuated the disruption of intestinal barrier integrity and the decreased expressions of ZO-1 and Occludin in the ileum, duodenum, and colon induced by HDOx. The abundance of *Ruminococcaceae_UCG-014* was found to be positive correlated with the expression levels of intestinal barrier indicators, including MUC2, Occludin, claudin-1, etc., in Ulcerative colitis (UC).^48^ Cao et al. also demonstrated that *Ruminiclostridium UCG-014* was negatively correlated with the gut barrier damage in mice.^49^ For *Parasutterella*, though there is no direct evidence about its effect on intestinal barrier, it has been reported to be associated with intestinal inflammation, including irritable bowel syndrome, intestinal chronic inflammation and Crohn’s disease. Chiodini et al. demonstrated that a significant accumulation of *Parasutterella* in the submucosa of the Crohn’s disease patients.^50^ Similarly, Chen et al. also found that the abundance of *Parasutterella* was positively associated with chronic inflammation caused by irritable bowel syndrome in various intestinal segments.^51^ As inflammation is one of the key reasons of intestinal barrier dysfunction, these results suggest that *Ruminiclostridium UCG-014* and *Parasutterella* may play important role in the regulation of intestinal barrier’s integrity. Furthermore, FMT effectively reversed the HDOx-induced decrease in the expressions of oxalate transporters, including Slc26a6 and Slc26a1, which are responsible for excreting oxalate into the intestinal lumen, particularly in the ileum.^47^ Interestingly, the expression of Slc26a3, which is predominantly expressed in the intestine and facilitates the absorption of oxalate from intestine, was not affected by HDOx nor FMT treatment. Moreover, FMT treatment led to a slight increase in fecal oxalate levels while significantly decreasing urinary oxalate levels. Nevertheless, further research is required to determine if oxalate or its precursor directly affects the expression of tight junction proteins. Overall, our findings indicate that FMT can attenuate HDOx-induced urinary oxalate excretion not only by suppressing oxalate intestinal absorption through the repair of damaged tight junctions but also by enhancing the intestinal secretory excretion of oxalate through increased expressions of oxalate transporters. These insights may contribute to the development of potential therapeutic strategies for hyperoxaluria.

However, some limitations in this study should be noticed. Though PICRUSt could provide valuable functional predictions of the microbiome, it is limited by its reliance on functional profiles derived from a small segment of a highly conserved gene. Future studies should consider metagenomics or metatranscriptomics to provide more comprehensive analysis about oxalate degradation enzymes. Additionally, *Ruminococcaceae_UCG-014* and *Parasutterella*, along with their metabolites, such as Elaidic acid, 2-Hydroxycinnamic acid and Arachidonic acid, were found to play key roles in HDOx-induced urinary oxalate excretion, kidney injury and CaOx crystal depositions, but their direct effects on oxalate degradation and kidney injury still have not been explored. Furthermore, there are interspecies differences between rat and human, only the disrupted gut microbiota and metabolism in HDOx-induced hyperoxaluria rats were analyzed, further study is needed to determine the effects in human samples. Moreover, FMT interventions were performed throughout the trial period, but the specific times when the FMT works had not been established. The clarification of these questions will contribute to a more accurate treatment of hyperoxaluria using FMT.

## Conclusion

**Figure 6.**
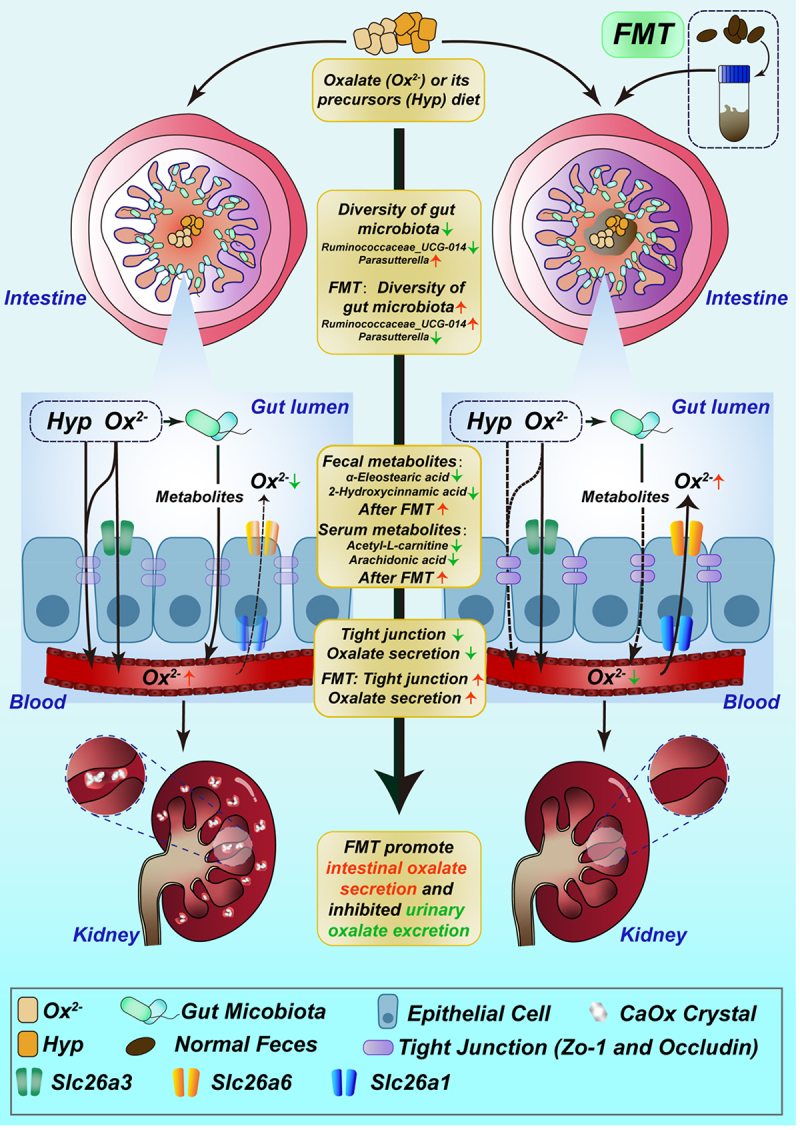
Schematic diagram of FMT attenuating HDOx-induced hyperoxaluria.

In conclusion, our study provides significant contributions to the understanding of the complex interplay between gut microbiota, metabolites, and oxalate metabolism in the context of high dietary oxalate (HDOx) intake and the development of secondary hyperoxaluria. Our comprehensive investigation revealed that HDOx exposure induces notable disturbances in gut microbiota composition and perturbs specific metabolites, ultimately leading to compromised gut function and dysregulated oxalate excretion in the urine. These interconnected alterations play pivotal roles in the pathogenesis of hyperoxaluria and the subsequent kidney injury and depositions of CaOx crystals. Remarkably, our correlation analysis highlights the potential roles of specific gut bacteria, particularly *Ruminococcaceae_UCG-014* and *Parasutterella*, along with their associated metabolites such as Elaidic acid, 2-Hydroxycinnamic acid, and Arachidonic acid, in mediating HDOx-induced hyperoxaluria, kidney injury and CaOx crystal depositions (Figure 6). This finding adds to the understanding of the mechanisms underlying HDOx-induced hyperoxaluria, emphasizing the importance of the gut-kidney axis in this process.

Overall, our study advances the understanding of the intricate relationship between gut microbiota, metabolites, and oxalate metabolism, shedding light on potential therapeutic targets for managing oxalate-related disorders, including hyperoxaluria and CaOx stone. The therapeutic potential of fecal microbiota transplantation (FMT) is underscored as a promising intervention to counteract HDOx-induced disorders by restoring gut microbiota composition and function. Further investigations are warranted to explore the direct impact of specific gut bacteria and metabolites on kidney injury and oxalate metabolism, and to bridge the translational gap for potential clinical applications. By optimizing the timing and duration of FMT interventions, this approach may offer new avenues for preventive and therapeutic strategies, ultimately improving clinical outcomes and enhancing the quality of life for individuals affected by oxalate-related disorders.

## Supplementary Material

Supplemental Material

## Data Availability

All data generated or analyzed during this study are included in this published article and its supplementary information files. Datasets not directly provided in the publication are available from the corresponding author upon reasonable request as stated in the Data Availability Statement.
