# Supplementary material for: Gut microbiota modulation via fecal microbiota transplantation mitigates hyperoxaluria and calcium oxalate crystal depositions induced by high oxalate diet
Source: Gut Microbes. 2025 Jan 28;17(1):2457490. doi: 10.1080/19490976.2025.2457490 (PMC11776474; doi:10.1080/19490976.2025.2457490)
Supplement: Supplemental Material [file KGMI_A_2457490_SM0143.zip › Supplement 2457490/Supplementary Figure Legend (1).doc]

Figure S1. The differential phylum between indicated groups

Statistical analysis of phyla across different groups over the time course. * P < 0.05, ** P < 0.01, *** P < 0.005.

Figure S2. The differential genera between indicated groups

(a) LEfSe analysis identified the most differentially abundant genera between the Normal group and the HDOx groups on day 45. (b) Genera Indicator value of the Normal group and HDOx groups at day 45 were analyzed using Indicator Species Analysis. (c) MaAslin was used to identified the differentially abundant genera of HDOx groups between the day 0 and day 45. (d) Genera Indicator value of genera in HDOx 0day and HDOx 45days were analyzed using Indicator Species Analysis. (e,f) The alteration trend of differential genera on day 0, 15, 30, 45.

Figure S3. The gut microbiota structure of rats on the day 45

(a) The differential genera between the Normal group (day 45) and the 3% K_2_Ox group (day 45). (b) The differential genera between the Normal group (day 45) and the 3% Hyp group (day 45). (c) The alpha diversity of gut microbiota on day 45, including Chao 1 and Shannon index. (d) The relative abundance of HDOx groups on day 45 at the phylum level. * P<0.05

Figure S4. The number of differential metabolites

(a) The number of fecal metabolites induced by HDOx in feces. (b) The number of fecal metabolites induced by HDOx in serum.

Figure S5. The excretion of 24-h urine on the timeline

(a) The fecal oxalate and the 24h urinary oxalate of each groups were detected at the day 45. (b) The 24-hr urine excretion of 3% K_2_Ox and its corresponding FMT groups. (c) The 24-hr urine excretion of 3% Hyp and its corresponding FMT groups. (d) The 24-h urine excretion of 5% K2Ox and its corresponding FMT groups. (e) The 24-h urine excretion of 5% Hyp and its corresponding FMT groups. * :P < 0.05 compared to the 0 day, # :P < 0.05 compared to day 15.

Figure S6. The improvement of FMT on gut microbiota disorders induced by HDOx

(a) FMT could improve the αlpha diversity of rat gut microbiota (Chao1 index and Shannon index) in the HDOx groups. (b) Effects of FMT on the beta diversity of gut microbiota analyzed by PCoA. * P < 0.05.

Figure S7. The effects of FMT on HDOx-induced differential bacteria (genus level)

(a) The relative abundance of common differential bacteria in the 5% K_2_Ox^FMT^ group compared to the 5% K_2_Ox group at day 45. (b) The relative abundance of common differential bacteria in the 5% Hyp^FMT^ group compared to the 5% Hyp group at day 45. (c) The relative abundance of differential bacteria in the 5% K_2_Ox^FMT^ group compared to the 5% K_2_Ox group at day 45. (d) The relative abundance of differential bacteria in the 5% Hyp^FMT^ group compared to the 5% Hyp group at day 45. * P < 0.05, ** P < 0.01, *** P < 0.005.

Figure S8. The relative abundance of oxalate degrading enzyme in different groups

(a) The relative abundance of oxalate degrading-related enzyme among the normal, 5% K_2_Ox, and 5% K_2_Ox^FMT^ group at day 45. (b) The relative abundance of oxalate degrading-related enzyme among the normal, 5% Hyp, and 5% Hyp^FMT^ group at day 45.

Figure S9. The effects of FMT on HDOx-induced intestinal oxalate transporters’ expressions

(a) The IF staining of oxalate transporters (Slc26a6, Slc26a1 and Slc26a3) in ileum and duodenum among different groups. (b) The IF staining of oxalate transporters (Slc26a6, Slc26a1 and Slc26a3) in colon among different groups. Bar = 100 μm.
