# Supplementary material for: Gut microbiota modulation via fecal microbiota transplantation mitigates hyperoxaluria and calcium oxalate crystal depositions induced by high oxalate diet
Source: Gut Microbes. 2025 Jan 28;17(1):2457490. doi: 10.1080/19490976.2025.2457490 (PMC11776474; doi:10.1080/19490976.2025.2457490)
Supplement: Supplemental Material [file KGMI_A_2457490_SM0143.zip › Supplement 2457490/Supplementary Table (2).doc]

**Table S1. Pairwise Adonis comparisons test of beta diversity analysis for each group**

| difference | R2 value | P value |
| --- | --- | --- |
| Normal-15day vs 3%Hyp-15day | 0.2984 | 0.001 |
| Normal-15day vs 5%Hyp-15day | 0.227 | 0.005 |
| 3%Hyp-15day vs 5%Hyp-15day | 0.0827 | 0.322 |
| Normal-15day vs 3%Hyp-15day vs 5%Hyp-15day | 0.2558 | 0.002 |
| Normal-30day vs 3%Hyp-30day | 0.1083 | 0.145 |
| Normal-30day vs 5%Hyp-30day | 0.1327 | 0.055 |
| 3%Hyp-30day vs 5%Hyp-30day | 0.0585 | 0.561 |
| Normal-30day vs 3%Hyp-30day vs 5%Hyp-30day | 0.1268 | 0.144 |
| Normal-45day vs 3%Hyp-45day | 0.2422 | 0.002 |
| Normal-45day vs 5%Hyp-45day | 0.2837 | 0.003 |
| 3%Hyp-45day vs 5%Hyp-45day | 0.3764 | 0.002 |
| Normal-45day vs 3%Hyp-45day vs 5%Hyp-45day | 0.3739 | 0.001 |
| Normal-15day vs 3%K_2_Ox-15day | 0.3741 | 0.001 |
| Normal-15day vs 5%K_2_Ox-15day | 0.5202 | 0.001 |
| 3%K_2_O-15day vs 5%K_2_O-15day | 0.1926 | 0.004 |
| Normal-15day vs 3%K_2_Ox-15day vs 5%K_2_Ox-15day | 0.4381 | 0.001 |
| Normal-30day vs 3%K_2_Ox-30day | 0.4554 | 0.001 |
| Normal-30day vs 5%K_2_Ox-30day | 0.4157 | 0.001 |
| 3%K_2_Ox-30day vs 5%K_2_Ox-30day | 0.0941 | 0.077 |
| Normal-30day vs 3%K_2_Ox-30day vs 5%K_2_Ox-30day | 0.4169 | 0.001 |
| Normal-45day vs 3%K_2_Ox-45day | 0.3368 | 0.002 |
| Normal-45day vs 5%K_2_Ox-45day | 0.4062 | 0.001 |
| 3%K_2_Ox-45day vs 5%K_2_Ox-45day | 0.1708 | 0.003 |
| Normal-45day vs 3%K_2_Ox-45day vs 5%K_2_Ox-45day | 0.3715 | 0.001 |

**Table S2. Pairwise Adonis comparisons test of metabolites for each group**

|  | difference | R2 value | P value |
| --- | --- | --- | --- |
| Feces | Normal-vs-3%K_2_Ox | 0.3199 | 0.001 |
|  | Normal-vs-5%K_2_Ox | 0.2532 | 0.004 |
|  | 3%K_2_Ox-vs-5%K_2_Ox | 0.1731 | 0.024 |
|  | Normal-vs-3%K_2_Ox-vs-5%K_2_Ox | 0.303 | 0.001 |
| Serum | Normal-vs-3%K_2_Ox | 0.2421 | 0.002 |
|  | Normal-vs-5%K_2_Ox | 0.2704 | 0.001 |
|  | 3%K_2_Ox-vs-5%K_2_Ox | 0.2198 | 0.005 |
|  | Normal-vs-3%K_2_Ox-vs-5%K_2_Ox | 0.301 | 0.001 |
| Feces | Normal-vs-3%HYP | 0.1335 | 0.044 |
|  | Normal-vs-5%HYP | 0.3846 | 0.002 |
|  | 3%HYP-3-vs-5%HYP | 0.3642 | 0.001 |
|  | Normal-vs-3%HYP-vs-5%HYP | 0.388 | 0.001 |
| Serum | Normal-vs-3%HYP | 0.2984 | 0.001 |
|  | Normal-vs-5%HYP | 0.5378 | 0.001 |
|  | 3%HYP-vs-5%HYP | 0.196 | 0.001 |
|  | Normal-vs-3%HYP-vs-5%HYP | 0.441 | 0.001 |
